# Hospital Meetings, &C.

**Published:** 1898-05-21

**Authors:** 


### HOSPITAL MEETINGS, &C.

### THE HOSPITALS ASSOCIATION.

The annual meeting of The Hospitals Association was held at the Hospital Building, 28 and 29, Southampton Street, Strand, W.C., on Friday, the 13th inst., Mr. H. Cosmo O. Bonsor, president of the association, in the chair.

The Chairman briefly moved the adoption of the report, in which it was stated that the council were glad to be able to report most satisfactory issues to two matters which had engaged their attention during the year 1896. First, the grievance under which the nurses laboured from "The Poor Law Officers' Superannuation Act, 1896," had been remedied by the passing of an amending Bill in the last Session of Parliament, and nurses had now the option of contracting out of the Act. Secondly, a Central Hospital Council had been successfully constituted by action on the part of the hospitals themselves in accordance with the principles laid down at the meeting of the association in April, 1896, at Charing Cross Hospital. The new council consisted of three representatives from each of the metropolitan hospitals having medical schools, under the chairmanship of Mr. J. G. Wainwright, the treasurer of St. Thomas's Hospital. Its composition was such as to command the confidence of the public, and it should prove, under proper guidance, a most useful body to all concerned in hospital work. Though so recently established, it had already won recognition in the highest quarters, as qualified to speak authoritatively on questions of hospital management, and the council of the association congratulated the hospitals on this happy termination to a prolonged controversy. After referring to the papers which had been read during the past session the report stated that the question of the rating of hospitals had been engaging the attention of the Council, and they had been in communication with the Charities Rating Exemption Society on the subject; but, after giving it exhaustive consideration, it had been decided to take no further action in this connection. The report of the Ambulance branch showed that, during the year 1897, 2,086 accident cases were removed by means of The Hospitals Association street ambulances, a compared with 1,684 in 1896, 1,791 in 1895, and 1,652 in 1894. The figures showed that the ambulances were more widely employed and were much more frequently used by the police than had hitherto been the case.

Sir Henry Burdett, in seconding, said that the chief points alluded to in the report illustrated in an excellent way the special objects for which the association was formed. First, they showed that when one large body of workers in the hospital world—the Poor Law nurses—was in trouble, and when the rights of this body had been interfered with by the action of Parliament, The Hospitals Association was utilised to form a neutral platform on which all parties could meet and where the differences between them could be thoroughly threshed out, and a satisfactory understanding arrived at. (Hear, hear.) Secondly, as a result of a resolution passed at the meeting of the association in April, 1896, the hospitals had now the pleasure of recognising and welcoming the appointment of the Central Hospital Council for London, whose utility and influence had already been proved by the circumstance that a reference had been made to it by the Prince of Wales's Fund, which had requested it to advise the Fund as to what hospitals were conducted upon proper principles, and so were entitled to receive a grant from the Fund. There could be no doubt that a Central Board of any kind to be of any use to anyone must be representative of the interests and must consist of men who were familiar with the details of the work of all the institutions which it was proposed to put under its control. (Hear, hear.) Therefore, it was a matter for congratulation that London had in the Central Hospital Council a body which had the confidence of all engaged in hospital work, and which was entitled to the confidence of the public and the support of the hospitals. Another point which he thought was worthy of being brought out was the fact that The Hospitals Association existed for the purpose of enabling matters which are not quite ripe enough to be dealt with in a practical form by individual hospitals to be fully discussed. The conductors of the association had not held unnecessary meetings, nor had they unnecessarily worried with subjects which were either not ripe for discussion or were not of immediate public interest. That was a policy which he ventured to hope would be continued by the association, for he was certain that whilst it was desirable that the association should be maintained and kept together as a neutral platform, it would be undesirable that that platform should be utilised by fussy people who had more time than wisdom to bring to bear upon the subjects to be discussed. Another portion of the association's work was the street ambulance branch, about which the public knew far too little, owing to the fact that it was entirely maintained by the treasurer, Mr. H. L. Bischoffsheim—(applause)—who desired above all things to keep his identity as much as possible in the background. All Mr. Bischoffsheim had desired to do was to provide that the old bad system by which a man or a woman when overtaken by accident in the public streets of London was hustled into a cab, which often resulted in increasing the injuries of the patient, should be entirely obviated. He (the speaker) was perfectly clear that London owed a debt of real gratitude to Mr. Bischoffsheim, because through the street ambulance branch of this association there were placed in every crowded part of London ambulances with which the police were thoroughly familiar, and in which from two-thirds to three-quarters of the street accidents every year were conveyed to the hospitals. (Applause.) No small part of the success of the association's street ambulance system was due to the continuous supervision of Mr. Thomas Ryan, the hon. secretary. (Applause.)

The report was unanimously adopted.

On the motion of Sir Henry Burdett, seconded by Mr. F. D. Mocatta, Mr. H. Cosmo O. Bonsor was re-elected president for the ensuing year. The Council and other officials were also re-elected, and the meeting terminated with a vote of thanks to the chairman.

### CITY OF LONDON HOSPITAL FOR DISEASES OF THE CHEST.

The forty-fourth festival dinner which celebrates the fiftieth anniversary of the establishment of the City of London Hospital for Diseases of the Chest was held on Thursday, the 12th inst., at the Hotel Cecil. The Duke of Connaught, who was attended by Colonel Egerton, presided, and was supported by the Duke of Leeds, the Earl of Denbigh, Sir Edward Sassoon, Bart., Sir W. Corry, Bart., Sir Arthur Cowell-Stepney, Bart., Sir M. M. Bhownaggree, M.P., Mr. Spencer Charrington, M.P., Mr. S. Herbert Robertson, M.P., Colonel Hozier, C.B., and others,

After the usual loyal toasts had been proposed,

Mr. Herbert Robertson, M.P., in responding for the "Houses of Parliament" (proposed by Mr. J. Compton), said there were various ways in which the Houses of Parliament could come directly into contact with hospitals. In the first place they might pass legislation affecting hospitals. People were cynical enough to say that the less legislation on any subject the better. (Laughter.) Without in any way expressing an opinion on that point he would say that the less legislation there was affecting hospitals, or dealing with them at all, and the less Parliament interfered with those gentlemen who managed or supported hospitals, the better for the hospitals. In the second place Parliament might contribute to hospitals out of the pocket of the nation. There were many who thought that would be a very good thing, but personally he did not think so, as it would check the flow of public charity to the hospitals, and would very much cripple their efforts. (Hear, hear.)

The Chairman, who was received with great enthusiasm, proposed the toast, "Prosperity to the Hospital." He said that when they considered what the hospital had done during the fifty years of its existence they had reason to be satisfied with its good work, for during that period no less than 31,000 in- and 590,000 out-patients had been treated. The hospital was started in the year 1848 to meet a great want in the East of London, namely, to treat those diseases of the chest and heart which were, alas! so prevalent in that part of the metropolis owing to the work on which the inhabitants were employed and to the surroundings in which they lived. The foundation-stone was laid by Prince Consort—(loud applause)—and Her Majesty the Queen—(renewed applause)—and during the existence of the hospital Her Majesty had lost no occasion of showing the deep interest she had taken in the hospital. (Applause.) Referring to the closed beds, his Royal Highness said that it was only since last June that it had been found necessary to close 120 beds out of the 164 available. Since then there had been a slight reduction, and at the present time there were only 80 beds unopened. (Hear, hear.) The fiftieth anniversary of this hospital might well be marked by an attempt to rectify this misfortune. To show how necessary this hospital was considered to be by working men, he might tell his hearers that they had been liberal subscribers, and they had this year, out of their small and hard-gained earnings, promised subscriptions amounting to £1,100. Surely when working men had come forward in this generous manner, those who were richer and more able to give would not be behindhand. (Applause.) He hoped that the result of that dinner would be a thoroughly satisfactory one, and that next year they would have the satisfaction of knowing that this hospital had been opened in its entirety. He did not appeal for funds to build any new wards, but only for funds to enable those which existed to carry out all the work they were capable of doing. (Loud applause.)

Other toasts followed.

Mr. J. Benson (as chairman of the committee), in responding for the officials, announced that Sir M. M. Bhownaggree had requested the committee to accept from him a bronze bust of Her Majesty, to be erected in the grounds of the hospital as a memorial of Her Majesty's Diamond Jubilee.

The Secretary announced donations and subscriptions amounting to £2,866, the largest sum received at a festival dinner in aid of the hospital since that at which the Duke, as Prince Arthur, presided 26 years ago.

### THE FREE HOME FOR THE DYING.

The annual gen[illegible]ral meeting of the governors of the Free Home for the Dy[illegible]g, 82, The Chase, Clapham, S.W., was held at the Churc[illegible] House, the [illegible]ean's Yard, Westminster, at four o'clock on Mon[illegible]ay. There were present the Chairman (the Rev. C. E. Brooke), Lieut.-Colonel Barrington-Foote, R.A., the Hon. P. Thesiger, C.B., Colonel J. P. Ellis Jervaise, Mr. W. Hoare (the treasurer), Captain W. Portlock-Dodson and Miss Clifford (secretaries), and the Sister in charge. As a a copy of the report had been sent to each governor, it was taken as read and accepted. The members of the council were re-elected, and Mr. Hoare was added thereto. The officers were re-elected, and Messrs. Lovelock and Linnet appointed professional auditors for the ensuing year. The bye-laws were read and approved, and the meeting terminated. The income for the year was £1,410, and in spite of the erection of a furnace to destroy dressings, &c., there is a balance to the good of £196. During the year 48 patients were admitted; 30 died, nine were discharged, and nine remained.

### NORTH EASTERN HOSPITAL FOR CHILDREN.

Mr. Walter Johnson, who presided at the annual meeting of this institution on Monday, remarked that the report and accounts were on the whole satisfactory, and he was glad to be able to add that sufficient progress had been made with the building fund to warrant steps being taken in regard to the erection of the new wing to complete the hospital. On this point the report remarks that the interest of all who know the hospital is now centered in the completion of its building; and the committee welcome the co-operation of the large and influential local committee which has been formed amongst the members of the vestries and boards of guardians of Shoreditch, Bethnal Green, and Hackney and other residents in the neighbourhood for the purpose of supporting their appeal and emphasising the need that exists for increased accommodation in every department of the hospital. The income of 1897 amounted to £5,683, and the total expenditure to £5,717, leaving a trifling deficiency. The annual subscriptions show a steady increase, but no legacies were received during the year. In the course of 1897, 772 patients were admitted to the wards, as against 705 in the year preceding. Of these 455 were cured, 156 relieved, and 50 unrelieved, while 111 died. In the out-patient department, the cases numbered 14,997, and the attendances, including accidents, 54,352. After exhaustive inquiry, it was decided early in 1897 to open a special department for dentistry, and during the nine months 755 patients attended. The committee have been giving attention to the question of out-patient abuse, and have decided to institute a system of inquiry under proper safeguards, with a view to preventing persons well able to afford the fees of a general practitioner from receiving the benefits of the hospital. In the course of his speech, the Chairman observed that the death of the late president, Mr. Joseph Gurney Barclay, was so recent, that no steps had been taken to replace him, no easy task when they recalled his munificent help to the hospital. A committee, however, would be called, if necessary, to arrange for the election of a suitable president. Mr. Johnson also stated arrangements were being made for the installation of the electric light throughout the hospital, and the Secretary added that the Röntgen rays apparatus was also being introduced.

